# Block-And-Lock Strategies to Cure HIV Infection

**DOI:** 10.3390/v12010084

**Published:** 2020-01-10

**Authors:** Gerlinde Vansant, Anne Bruggemans, Julie Janssens, Zeger Debyser

**Affiliations:** Laboratory for Molecular Virology and Gene Therapy, Department of Pharmaceutical and Pharmacological Sciences, Katholieke Universiteit, Leuven, 3000 Flanders, Belgium

**Keywords:** HIV, latency, cure, block-and-lock

## Abstract

Today HIV infection cannot be cured due to the presence of a reservoir of latently infected cells inducing a viral rebound upon treatment interruption. Hence, the latent reservoir is considered as the major barrier for an HIV cure. So far, efforts to completely eradicate the reservoir via a shock-and-kill approach have proven difficult and unsuccessful. Therefore, more research has been done recently on an alternative block-and-lock functional cure strategy. In contrast to the shock-and-kill strategy that aims to eradicate the entire reservoir, block-and-lock aims to permanently silence all proviruses, even after treatment interruption. HIV silencing can be achieved by targeting different factors of the transcription machinery. In this review, we first describe the underlying mechanisms of HIV transcription and silencing. Next, we give an overview of the different block-and-lock strategies under investigation.

## 1. Introduction

Despite significant improvements in clinical outcome, the HIV/AIDS pandemic remains an important threat to public health. Although combination antiretroviral therapy (cART) suppresses plasma viral load to undetectable levels, removal of therapy leads to a viral rebound from a highly stable reservoir of latently infected cells [1]. This reservoir mainly consists of resting memory CD4 T cells and can be found in many different anatomical compartments such as brain, liver, bone marrow and lymphoid tissues [2]. These latently infected cells escape the immune system and are not eliminated by current antiretroviral treatments [3]. Hence, the persistence of these latent reservoirs is the major obstacle towards a cure for HIV-1 infection.

The potential for an HIV cure was highlighted by the long-term HIV remission of two infected individuals (the Berlin and London patient) following an allogeneic stem cell for either leukemia or lymphoma, respectively [4,5]. Both patients received stem cell transplants from donors with a homozygous CCR5Δ32 mutation, rendering the resulting CD4+ T cells resistant to HIV infection by R-tropic strains that use the CCR5 co-receptor for infection. Notably, another patient treated with such CCR5Δ32 stem cells suffered viral rebound from a minority X-tropic strain, which uses the CXCR4 co-receptor, in his reservoir [6,7]. Other patients who received allogeneic stem cell transplantations lacking this mutation rebounded as well [8]. In short, the significant mortality risk, the low chance of finding a HLA-matching donor with CCR5Δ32 and the possibility of rebound even with such a donor mean this treatment is not scalable for the vast majority of HIV-infected individuals. Significant effort has been directed towards the development of potential cures that eliminate the latent reservoir. Studies are ongoing to remove HIV-1 provirus from latent cells using gene-editing strategies [9,10,11]. However, delivery of gene editing constructs to all reservoir cells in vivo remains a formidable hurdle and gene-editing strategies suffer from unknown off-target risks [12].

Alternatively, the shock-and-kill strategy aims to eradicate the reservoir by repeated reactivation of latent cells that are subsequently killed by the immune system or viral cytopathic effects [13]. Initial clinical trials with several latency reversing agents (LRAs) showed induction of viral RNA production in patients, e.g., by disulfiram and the HDAC inhibitors vorinostat, panobinostat or romidepsin. However, these LRAs did not reduce the size of the latent reservoir [14,15,16]. Besides low efficacy in the clinic, other limitations of many LRAs are their side effects and toxicity by affecting cellular homeostasis. Moreover, studies show that only a minor fraction of the reservoir is reactivated upon treatment with LRAs, indicating that a combination of multiple LRAs is required [17,18]. Combination approaches, in which LRAs from multiple mechanistic classes are combined, are now investigated to obtain a more effective shock [19,20,21]. Still, reactivation of latently infected cells is not sufficient to reduce the size of the reservoir. Shan et al. showed in a primary cell model that latently infected cells survive despite viral cytopathic effects and the presence of cytotoxic T cells [22]. The infected cells were only killed upon antigen-specific stimulation of the cytotoxic T cells [22]. Therefore, the ‘kill’ phase requires optimization by improving immune responses and stimulating apoptosis of infected cells [23,24]. The immune response can be stimulated by TLR agonists [25], immune checkpoint inhibitors [26], therapeutic vaccines [27] and broadly neutralizing antibodies [28,29]. Currently several pro-apoptotic compounds are tested for their capacity to kill latently infected cells, e.g., SMAC (second mitochondria-derived activator of caspase) mimetics [30,31,32] and inhibitors of the regulator protein B cell lymphoma 2 (Bcl2) [33,34] and PI3K/Akt pathway [35].

The limited success of eradication strategies has caused scientists and clinicians to re-evaluate the definition of HIV cure. The ultimate outcome would indeed be the complete eradication of all replication-competent HIV. However, such a sterilizing cure will be challenging to achieve. A more feasible outcome might be long-term HIV remission or a functional cure. A functional cure could be achieved by durably silencing the latent provirus in infected cells and thereby preventing viral rebound [36]. This so-called block-and-lock strategy prevents HIV transcription and reactivation in latently infected cells. In this review, we will first discuss the HIV transcriptional machinery and determinants leading to transcriptional silencing. Secondly, we will give an overview of various block-and-lock HIV cure strategies acting on different determinants of HIV transcription.

## 2. HIV Transcription and Silencing

Viral latency is classified in two forms, based on the integration status of the viral DNA: pre- and post-integration latency. Pre-integration latency occurs when the replication cycle is interrupted prior to integration [37]. Unintegrated DNA can be linear or circularized, but generally has a short half-life of approximately one day [38]. Although they may persist longer in macrophages or the brain, they are clinically less relevant [39,40]. Post-integration latency takes place when the virus is stably integrated in the host genome but fails to express proteins. As such, the provirus can persist for the lifespan of the infected cell. Post-integration latency can be sustained by blocks in nuclear export of viral RNA and translation, but often HIV is silenced at the transcriptional level. In this review, we will elaborate on HIV transcription as many of the current block-and-lock strategies act on various determinants of HIV transcription.

**Figure 1:**
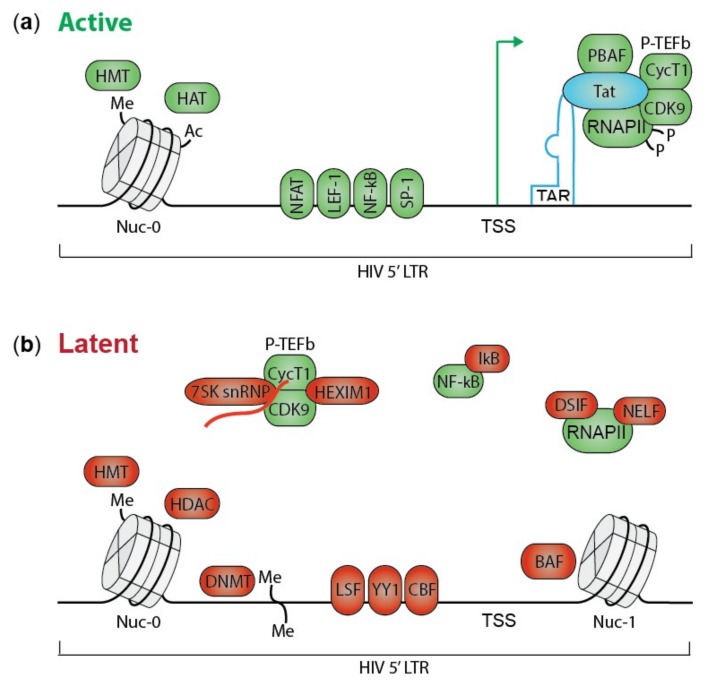
The HIV LTR promoter in active and latent state. (**a**) HIV Tat induces active transcription by binding the TAR RNA element in the LTR promoter and recruiting several transcription activating proteins. P-TEFb releases and activates RNAPII by CDK9-mediated phosphorylation. PBAF ensures open nucleosome-free chromatin. NF-κB and other transcription factors as NFAT, LEF-1 and SP-1 bind to the HIV LTR. An active chromatin landscape is maintained by histone methyl transferases (HMT) and histone acetyl transferases (HAT). (**b**) In latent cells, HIV transcription is inhibited by multiple mechanisms. Several proteins required for effective transcription are sequestered in an inactive state. For instance RNAPII is sequestered by DSIF and NELF, NF-κB by IκB, and P-TEFb by 7SK snRNP and HEXIM1. Furthermore, transcriptional repressors as LSF, YY1 and CBF bind the LTR promoter. A repressive chromatin landscape is formed by HMT, histone deacetylases (HDAC) and DNA methyl transferases (DNMT). Finally, BAF positions Nuc-1 downstream of the TSS, inhibiting transcription elongation. Tat; transactivator of transcription, TAR; transactivation response RNA, P-TEFb; positive transcription elongation factor b, RNAPII; RNA polymerase II, CDK9; cyclin dependent kinase 9, PBAF; polybromo-associated BAF, NF-κB; nuclear factor kappa b, NFAT; nuclear factor of activated cells, LEF-1; lymphoid enhancer-binding factor 1, SP-1; specificity protein 1, HMT; histone methyl transferase, HAT; histone acetyl transferase, DSIF; DRB sensitivity inducing factor, NELF; negative elongation factor, IκB; inhibitors of NF-κB, 7SK snRNP; 7SK small nuclear RNA, HEXIM-1; Hexamethylene bisacetamide-induced protein, LSF; late SV40 factor, YY1; yin yang 1, CBF; C-promoter binding factor, LTR; long terminal repeat, HDAC; histone deacetylase, DNMT; DNA methyl transferase, BAF; BRG-associated factor, Nuc-1; nucleosome 1, TSS; transcription start site. Green symbols represent factors promoting active transcription, while red symbols are transcriptional repressors.

HIV transcription is a complex machinery involving many viral and cellular factors. During productive infection, HIV initially produces short completely spliced transcripts encoding transactivator of transcription (Tat) and regulator of virion expression (Rev). When the amount of Tat protein reaches a certain threshold, it binds to the TAR RNA stem loop in the HIV LTR promoter stimulating transcription elongation (Figure 1a) [41]. Tat recruits the positive transcription elongation factor complex (P-TEFb), consisting of cyclin-dependent kinase 9 (CDK9) and Cyclin T1, to the transcription start site (TSS) in the LTR promoter [42]. Next, CDK9 releases and activates RNA polymerase II (RNAPII) that is sequestered by negative elongation factor (NELF) and DRB sensitivity inducing factors (DSIF) [43]. Furthermore, Tat recruits the active chromatin remodeling PBAF (polybromo-associated BRG-associated factor) complex promoting open chromatin [44]. Changes in these factors can silence HIV transcription resulting in latent infections.

Suboptimal concentrations or post-translational modifications of Tat hamper Tat-induced transcription elongation. For instance, phosphorylation of Tat by CDK2 results in inhibition of transcription [45]. Moreover, in latent cells P-TEFb is retained in an inactive form by hexamethylene bisacetamide-induced protein (HEXIM-1) and 7SK small nuclear RNA (7SK snRNA) (Figure 1b) [46,47]. Tat and bromodomain containing protein 4 (BRD4) can release P-TEFb by disrupting the inactive complex [48]. BRD4 represses HIV transcription by competing with Tat for the P-TEFb binding site [48,49].

Transcription is also affected by the absence or presence of host transcription factors and transcription repressors. Nuclear factor kappa B (NF-κB) is a transcription factor involved in T cell activation that activates HIV transcription even in the absence of Tat [50]. However, in resting cells NF-κB is sequestered in the cytoplasm by inhibitors of NF-κB (IκB). Nuclear factor of activated T-cells (NFAT), another transcription factor, is phosphorylated and resides in the cytoplasm of resting cells [51,52]. NF-κB and NFAT are two key factors for initiation of HIV transcription; for more examples of transcription factors involved in HIV latency please see following review articles [53,54,55,56]. Additionally, some repressive host factors like yin yang 1 (YY1), late SV40 factor (LSF) [57] and C-promoter binding factor (CBF) [58] recognize binding sites in the LTR promoter and limit transcription by recruiting histone deacetylases (HDAC) (Figure 1b) [59].

The chromatin and epigenetic landscape also affects HIV transcription [60,61]. The chromatin structure is defined by the presence of nucleosomes, consisting of eight core histones that can be epigenetically modified. Histone acetylation, induced by histone acetyl transferases (HATs), is associated with active transcription; acetylation loosens the chromatin making it more accessible to interacting proteins [62]. H3K36me3 is typically found in the body of active genes [63], while tri-methylation of H3K27 and H3K9, established by histone methyl transferases (HMTs), are associated with transcriptional silencing [64]. Additionally, DNA methylation at CpG dinucleotides in the LTR promoter contributes to latency and restricts reactivation in cell lines and patient samples by hampering the access of transcription factors to the DNA [65,66]. Regardless of the site of integration, the viral LTR promoter is occupied by two nucleosomes, nuc-0 and nuc-1. Nuc-1 is positioned downstream of the transcription start site (TSS) by the ATP-dependent chromatin remodeler BAF (BRG1-associated factor), where it blocks transcription elongation in latent cells (Figure 1b) [67].

Next to a block in transcription initiation or elongation, additional blocks may exist at the level of distal transcription and multiple splicing as postulated by the Yukl group [68]. They used reverse transcription droplet digital PCR to quantify different HIV transcripts in patient-derived cells. Short TAR RNA was most abundant, followed by RNA elongated beyond the LTR promoter, full length poly-adenylated RNA and finally multiple spliced RNA was the least common [68]. Interestingly, the transcription profiles differed in cells from blood compared to gut [69]. These results indicate blocks at transcription elongation, completion and RNA splicing that are tissue specific and might be important in HIV latency. Therefore, all steps involved in viral gene expression require further investigation.

The three-dimensional nuclear organization of chromatin affects the function of the DNA as well [70]. The genome organization within the nucleus is not random; transcriptionally active and inactive regions are physically distinguishable as decondensed euchromatin and condensed heterochromatin, respectively [71]. HIV preferentially integrates in open chromatin close to the nuclear pore, while heterochromatic chromatin in lamina-associated domains (LADs) or the inner nucleus is disfavored [72]. Moreover, nuclear processes like transcription, DNA-replication and -repair are located in certain structural compartments [70]. These facts indicate that the nuclear topography of HIV affects viral transcription.

Finally, the HIV integration site might be an important determinant of viral transcription as it defines both the epigenetic landscape and the 3D nuclear localization of the eventual provirus. HIV integration is not random; the virus has evolved in such a way that it hijacks cellular cofactors to tether its integration to active transcription units [73,74]. Depletion of the cofactors lens epithelium derived growth factor (LEDGF/p75) and cleavage and polyadenylation specificity factor 6 (CPSF6) were shown to decrease integration in active genes [75,76,77,78,79,80]. Moreover, proviruses that were retargeted by interfering with LEDGF/p75 were more latent and refractory to reactivation [76]. Finally, several studies suggest that integration in certain (onco-)genes supports clonal expansion of the infected cell contributing to viral persistence [81,82,83,84,85,86].

## 3. Block-And-Lock Strategies

Many viral and cellular proteins are involved in HIV transcription and silencing, and hence represent potential targets for future block-and-lock approaches. Several research groups have described mechanisms acting on different factors of HIV transcription in light of a block-and-lock strategy. We list these different block-and-lock strategies below.

### 3.1. Tat Inhibition by Didehydro-Cortistatin A

Currently the most advanced block-and-lock approach employs a Tat inhibitor, didehydro-cortistatin A (dCA), to silence HIV transcription. The viral Tat protein is an important factor for stimulation of HIV transcriptional elongation by recruiting and activating RNAPII (Figure 1) [87]. HIV Tat represents an interesting target since it is the first viral protein to be expressed upon infection and it has no cellular homolog. dCA is a potent Tat inhibitor that blocks HIV transcription and reactivation by different LRAs in cell lines and primary CD4+ T cells [88,89]. In 2017, the Valente lab used patient-derived cell models and bone marrow/liver/thymus (BLT) mouse latency models to show that prior treatment with dCA delayed and reduced viral rebound [90]. The BLT mice were co-treated with dCA and ART for four weeks prior to treatment interruption. Ten days later, all eight control mice displayed viremia, while the dCA treated mice showed a viral rebound only at day 19. Furthermore, dCA induced a high nucleosomal occupancy at the Nuc-1 region of the LTR promoter potentially explaining its long term effects [90]. In 2019 the Valente group elaborated on this mechanism by showing that dCA promotes tight nucleosome/DNA association by increasing deacetylated histone 3 occupancy at Nuc-1 [91]. Moreover, dCA enhanced the recruitment of the repressive BAF complex while the activating chromatin remodeling complex PBAF was inhibited. In line with these results, less RNAPII was detected at the transcription start site, even upon stimulation with LRAs. The specificity of dCA for Tat was confirmed by the lack of effect on Tat-TAR defective proviruses [91]. Altogether, these results show that dCA inhibits Tat-dependent transcription and induces a repressive epigenetic landscape that hampers HIV reactivation upon treatment interruption.

### 3.2. LEDGINs

In 2010, structure-based drug design identified the first small molecule inhibitors of the interaction between HIV integrase (IN) and the cellular chromatin-thethering factor LEDGF/p75 [92]. Inhibitors belonging to this class of antivirals [92,93,94,95,96], named ‘LEDGINs’, are unique due to their multimodal mechanism of action affecting both early and late stages of HIV-1 replication. LEDGINs inhibit HIV-1 integration and allosterically inhibit IN catalytic activity [92,97]. Moreover, LEDGINs enhance IN oligomerization during late stages of the replication cycle resulting in defective progeny virions [98,99,100,101]. Viral particles produced in the presence of LEDGINs display morphological defects and are less infectious [98,99,100,101]. The idea of using LEDGINs for a functional HIV cure arose when the Debyser lab started investigating their effect on integration sites and latency in 2016 [76,102]. Vranckx et al. showed that viruses capable of integrating in the presence of LEDGIN treatment during infection were retargeted out of active genes. The 3D localization of the provirus was closer to the inner nucleus [76]. Moreover, by using several reporter viruses and cell lines, they showed that these retargeted proviruses were more often in a latent state and refractory to reactivation by LRAs. These results were also confirmed in primary cells [76]. More recently, Vansant et al. showed that infection of cells with virus produced in the presence of LEDGINs also resulted in provirus with a more latent phenotype [103]. Based on these data, the Debyser group postulated that LEDGINs might be useful in a block-and-lock strategy by inhibiting viral integration and retargeting residual proviruses that manage to integrate in the presence of LEDGINs to sites that are less susceptible to reactivation [102]. In such a cure strategy, ideally LEDGINs are administered as soon as possible after acute infection to affect the formation of the reservoir. Although initial infection represents an interesting niche with over 1.7 million newly infected patients in 2018 [104], it is unclear whether a functional cure strategy with LEDGINs will also be useful in chronically infected patients for instance after treatment interruption. Before this concept moves on to clinically more relevant models such as humanized mice and eventually patients, further research on the mechanism of action of LEDGINs, specifically in the context of HIV latency, is required.

### 3.3. FACT Inhibition by Curaxin CBL0100

Another regulator of HIV transcription is the ‘facilitates chromatin transcription complex’ (FACT) that consists of suppressor of Ty16 (SUPT16H) and structure-specific recognition protein (SSRP1) [105]. FACT acts as a histone chaperone and promotes RNAPII driven transcription by destabilizing the nucleosomal structure [106]. In 2011, Gasparian et al. showed that the anticancer compounds named curaxins inhibit FACT and suppress NF-κB mediated transcription [107]. This finding led to the hypothesis that curaxins can promote HIV latency via inhibition of FACT. Indeed, in 2017 Jean et al. was able to block HIV replication and reactivation by using curaxin CBL0100 [108]. There was less reactivation of latent provirus, both in cell lines and in primary cell models, in the presence of CBL0100 compared to reactivation in the presence of DMSO. Curaxin CBL0100 inhibited RNAPII mediated transcription elongation in a Tat-dependent manner [108]. However, the effect was independent of NF-κB binding to the 5′ LTR promoter in contrast to its anti-tumor activity reported before [107,108]. The authors hypothesize that addition of CBL0100 to cART regimens might lead to a faster control of viremia and reduced HIV reactivation that eventually locks the virus in a latent state even upon treatment interruption. 

### 3.4. RNA-Induced Epigenetic Silencing

An alternative approach to silence HIV transcription is by using short interfering (si) or short hairpin (sh) RNA to maintain the repressive heterochromatic landscape at the HIV 5′ LTR promoter. The Kelleher group designed two siRNAs, 143 and Prom A, which target transcription factor binding sites in the LTR promoter [109,110]. siRNA 143 binds upstream of Nuc-0 where binding sites for transcription factors AP-1 (activator protein 1) and COUP (chicken ovalbumin upstream promoter) are located. siRNA Prom A targets a unique NF-κB binding site situated between Nuc-0 and Nuc-1. These siRNAs epigenetically silence HIV transcription by recruiting Argonaute 1 (AGO1), histone deacetylase 1 and histone methyl transferases [111]. AGO1 is an essential component of the RNA-induced silencing complex (RISC) that binds siRNAs and cleaves the mRNA, a process termed RNA interference (RNAi). Both siRNAs reduced reactivation of the latently infected J-Lat cells by two- to three-fold when challenged by different LRAs [111]. Thus, transcriptional gene silencing by siRNAs might be useful in a HIV block-and-lock functional cure via a gene therapy application. The siRNAs could be delivered to cART-treated patients via retroviral vector transduced autologous CD4+ T cells or CD34+ cells in the absence of cART [110]. However, further extensive preclinical evaluation is required.

### 3.5. HSP90 Inhibitors

Heat shock protein 90 (HSP90) is a cellular chaperone protein that helps folding and stabilizing other proteins. Heat shock proteins protect cells when stressed by high temperatures. They are also required for the production of viral proteins. Upon HIV infection, the expression of HSP90 increases in mononuclear cells and T cells [112,113]. Indeed, HSP90 inhibitors suppress HIV transcription and replication [114,115]. Moreover, HSP90 is involved in HIV reactivation by stimulating Tat-mediated HIV transcription and NF-κB, NFAT and STAT5 (signal transducer and activator of transcription) signaling [116,117]. Hyperthermia also enhances HIV transcription and reactivation [114,118]. Multiple HSP90 inhibitors have been reported to suppress HIV-1: GV1001, a peptide vaccine designed to induce T cell immunity, and specific HSP90 inhibitors such as AUY922 and 17-AAG [115,116,117,119]. AUY922 and 17-AAG are in clinical development as anticancer compounds [120,121,122,123]. In 2016, Joshi et al. showed that humanized BLT mice pretreated with a reverse transcriptase inhibitor (EFdA) and AUY922 or 17-AAG did not rebound up to 11 weeks after treatment cessation [119]. Upon heat shock or activation of the cells, replication competent virus was recovered from PBMC’s and the spleen, indicating that these cells were latently infected [119]. Thus, addition of HSP90 inhibitors to current treatment regimens might lead to long-term remission and potentially a functional cure of HIV infection.

### 3.6. Jak-STAT Inhibitors

Homeostasis of memory T cells, the major contributor of the latent reservoir, is regulated by cytokines that activate the Jak (Janus kinase)-STAT pathway. The Jak-STAT pathway was shown to be involved in HIV persistence and reactivation as two FDA approved Jak inhibitors, ruxolitinib and tofacitinib, were able to block HIV reactivation in primary CD4+ T cells [124]. Ruxolitinib and tofacitinib are approved for the treatment of hematologic conditions (myelofibrosis and polycythemia vera) and auto-immune conditions (rheumatoid arthritis, psoriatic arthritis and ulcerative colitis) respectively, and have strong anti-inflammatory effects. Latent cells were pre-treated with the two inhibitors for 30 min followed by reactivation in the presence of the compounds for 24 h. Ruxolitinib displayed the strongest inhibitory effect with more than 50% inhibition of reactivation measured by intracellular viral p24 [124]. Moreover, the anti-inflammatory effect of the Jak inhibitors reduced activation of T cells limiting transmission of HIV to other cells. Additionally, they reduced surface expression of the CCR5 co-receptor and decreased the number of CD4+ T cells harboring HIV provirus [125]. Currently, the anti-inflammatory effects of ruxolitinib in HIV infection and seeding of HIV reservoirs are evaluated in a phase 2 clinical trial (NCT02475655).

### 3.7. BRD4 Modulators

Another important regulator of HIV transcription is the bromodomain-containing protein 4 (BRD4). BRD4 is an epigenetic reader that interacts with various proteins to stimulate gene expression [48,126]. On the other hand, BRD4 inhibits HIV transcription by competing with Tat for binding to the P-TEFb [49]. Recently, Niu et al. identified a small molecule, ZL0580, that binds bromodomain 1 of BRD4 and suppresses HIV transcription [127]. ZL0580 inhibited Tat transactivation and transcription elongation. Additionally, the BRD4 modulator induced a repressive chromatin environment at the LTR promoter [127]. The small molecule delayed time to viral rebound after therapy cessation in PBMCs from aviremic HIV-infected patients. PBMCs treated with ART alone rebounded HIV replication after 2.4 ± 1.3 days, while virus rebounded only after 15 ± 6.1 days in PBMCs treated with ART plus ZL0580 [127]. Moreover, ZL0580 blocked spontaneous HIV replication in PBMCs of aviremic patients that were not treated with ART and blocked PHA stimulated reactivation [127]. As such, BRD4 modulators represent a new class of compounds that can be used for a block-and-lock functional cure strategy. Since the small molecule delayed but not prevented viral rebound, the authors speculate that probably a combination of approaches will be required to durably silence all HIV-1.

### 3.8. mTOR Inhibitors

To identify novel mechanisms contributing to HIV latency, the Verdin lab performed a genome wide analysis with a shRNA screen [128,129]. They transduced a latent J-Lat cell line with a vector encoding a mCherry fluorescent protein and a shRNA library targeting each protein coding gene [130]. Cells stably transduced with the shRNA express mCherry. The J-Lat cells contain one integrated latent HIV provirus per cell that expresses green fluorescent protein (GFP) upon activation. By characterizing mCherry and GFP double fluorescent cells, they identified three pathways important for HIV latency: transforming growth factor β (TGF- β), actin remodeling and mammalian target of Rapamycin (mTOR) signaling [128]. These pathways are linked as mTOR acts downstream of TFG- β signaling and upstream of actin remodeling. Inhibition of mTOR suppressed HIV reactivation in primary CD4+ T cells and patient cells by downregulating CDK9 phosphorylation and impeding NF-κB signaling [128]. The authors suggest that mTOR inhibition together with other latency promoting agents such as Tat and HSP90 inhibitors might be useful in a future ‘block a lock’ functional cure.

### 3.9. Kinase Inhibitors

Starting from the hypothesis that signaling pathways play a central role in HIV latency, Vargas et al. screened a library of kinase inhibitors targeting a wide range of signaling pathways in the latent 24ST1NLESG cell line [131]. They screened the kinase inhibitors in the absence or presence of different LRAs and found 12 inhibitors that blocked HIV-1 reactivation irrespective of the used LRA [131]. The four most potent compounds are PF-3758309, danusertib, AZ628 and P276-00 that target PAK, Aurora, Raf and CDK kinases, respectively. These compounds had IC_50_ values ranging from 0.0001 to 9.4 μM for blocking latency reversal by different LRAs in 24ST1NLESG cells. Additionally, they inhibited latency reversal in resting CD4+ T cells from HIV-infected donors that were challenged by the anti-CD3/CD28 monoclonal antibody [131]. Further studies are ongoing to evaluate whether these inhibitors could be useful in a block-and-lock functional cure strategy.

### 3.10. Triptolide

Triptolide is a diterpenoid epoxide derived from a Chinese herb that possesses anti-inflammatory, immunosuppressive and anti-tumor activities [132]. In 2014, Wan et al. first reported on the antiviral activity of triptolide [133]. Triptolide inhibited HIV-1 replication in vitro at the level of viral transcription. More specifically, triptolide hampered Tat-induced LTR activation by stimulating proteasomal degradation of Tat [133]. Currently, the effect of triptolide on the HIV-1 reservoir is being tested in phase III clinical trials in treatment-naive HIV-infected patients (NCT02219672). Whether triptolide has a beneficial effect in chronically and latently infected patients still needs to be studied.

## 4. Discussion

As described in this review, HIV transcription and latency are driven by complex interactions between cellular and viral proteins. Along with a growing understanding of these mechanisms, the potential targets for a functional block-and-lock cure increase. To evaluate the potential of the block-and-lock strategies, we must consider various factors such as effectiveness, advantages, scalability, potential side effects and challenges. Since patients on antiretroviral therapy have nearly normal lifespans these days and the side effects of newer cART combinations are reduced, new treatment modalities will need to clear a high bar.

With regard to efficacy, an important factor is the duration of the achieved HIV remission. Ideally, such a remission would be lifelong. Even though the idea of a ‘block and lock’ strategy arose only recently, this review listed the growing body of evidence showing that HIV transcription can be significantly downregulated. Though clinical data is still lacking, all of the strategies above impeded viral transcription and reduced reactivation in the presence of various LRAs in cell lines and/or primary cell models. More advanced studies in BLT mice showed that the Tat inhibitor dCA delayed viral rebound up to 19 days [90], while HSP90 inhibitors could delay rebound up to 11 weeks [119]. Yet so far, none of the investigated strategies has led to complete, long term suppression of virus in all cell and/or animal models. As many of these compounds are still in the early days of development and testing within the HIV field, newer compounds with improved efficacy could still be developed. Then again, HIV transcription can be stimulated in Tat dependent and independent manners, meaning blocking only one transcription pathway may not be enough to completely silence all proviruses. It could be that, as for the shock-and-kill approach, a combination block-and-lock treatment will be needed. It remains to be investigated whether the compounds can be administered during initial infection in combination with cART for a short period of time (induction therapy) or whether repeated dosing will be required (maintenance therapy).

The block-and-lock strategy holds great promises for the HIV cure field. First, since the approach targets HIV transcription in general, it is likely to affect not only the replication competent, but also the translation competent reservoir [134]. As most proviruses in the patients are defective, they cannot produce infectious progeny viruses, yet some do produce viral proteins, contributing to immune activation [135,136]. Hence silencing both replication competent and defective proviruses may have a positive effect on the patients’ health. Secondly, though the idea of living with an inactivated retrovirus may be scary to patients, scientifically speaking, this is not unprecedented. In fact, simian immunodeficiency viruses, HIV’s closest relatives, are non-pathogenic in many cases, meaning they have adapted to not affect the lifespan of their host [137]. In addition, fossils of past retroviral infections are spread throughout the human genome as endogenous retroviruses, some of which have played critical roles in human evolution [138]. These precedents hint that taming HIV through a block-and-lock strategy may in the end be more feasible than full eradication.

Another major advantage of the block-and-lock strategy compared to other approaches such as gene therapy and allogeneic stem cell transplantation is the scalability. Most block-and-lock strategies are based on small molecules and/or existing compounds and many of them are likely to be administered in an ambulatory setting, resulting in a relatively low cost. Hence, they can be deployed in the limited resource settings that form the heart of the current HIV epidemic. In contrast, gene therapy and stem cell transplantation are more expensive and require highly specialized and advanced technologies. In the end, if any HIV cure is to affect the global HIV epidemic, we must insist that it can be made available to as many people living with HIV as possible, including those without access to highly advanced medical technology. To be scalable worldwide, a functional cure would have to be effective against a broad range of viral strains. dCA has been shown to bind Tat of subtypes A, B, C, D and E, but virus strains resistant to dCA have been developed in vitro [139,140]. LEDGINs also target a viral protein, HIV integrase. Even though LEDGINs are active against a broad range of HIV strains, specific integrase mutations can induce LEDGIN resistance [97].

Targeting a cellular protein could help to avoid the problem of viral diversity and resistance, but this may come at the cost of more side effects. To our knowledge, curaxin is only in early clinical development (NCT03727789) as an anticancer drug, so data on side effects are scarce. The HSP90 inhibitor AUY922 has been tested in phase I and phase II clinical trials, which showed fatigue, diarrhea and visual disturbances as side effects [120,121,122]. Trials with 17 AAG have shown hepatotoxicity, headaches and gastro-intestinal burden as potential side effects [122,123]. Ruxolitinib and tofacitinib are linked to hematological abnormalities and skin cancer. Additionally tocafitinib increases the risk of opportunistic infections and gastro-intestinal perforations [141,142]. Further, the use of kinase inhibitors is impeded by their lack of specificity leading to damage in myocytes [143]. Finally, triptolide was shown to have toxic effects on the reproductive tract [144,145]. Though serious side effects may be acceptable in the fields where these treatments are currently used (cancer, myeloproliferative disorders, auto-immune disease), the risk benefit ratio for using them in virally suppressed HIV-infected patients with a normal life span for obtaining a functional cure will be different. Much will depend on the duration of the remission induction phase of treatment and/or the frequency of these treatments if the remission is not lifelong and repeats are necessary. Still, the side effects mentioned above are minor compared to the risks associated with allogeneic stem cell transplantations.

As with all cure strategies, including shock-and-kill and gene editing, the main challenge lies in reaching and affecting each cell containing replication competent provirus. If not each infected cell is targeted, HIV replication will eventually rebound. Although it is challenging to permanently silence all provirus with a block-and-lock approach, extending the time to viral rebound from a few weeks to months or years may already lead to a significant benefit for the patient. This will reduce the need and costs for cART and visits to clinic, and hence improve the quality of life of HIV-infected patients. However, along with this strategy comes a new challenge: since HIV reactivation seems to be a stochastically regulated process, how can we reliably predict a safe interval wherein no viral rebound will occur so patients can maintain their undetectable and non-transmissible HIV status? To answer this question we will need new tools to predict and/or quickly detect viral rebound. For cure research in general, we need better diagnostic tests to evaluate HIV reservoir size.

Currently there is no satisfactory cure for HIV and any strategy that could help us obtain a cure is worth investigating. As for the block-and-lock strategy, the next step will be bringing more of these treatments to in vivo models and clinical trials to investigate their effect on the latent HIV reservoirs and potential side effects in vivo. Though 35 years of HIV research have not resulted in a cure yet, the medical and scientific advances made by the field are unprecedented. As our knowledge and technology progresses, new insights will eventually lead to new breakthroughs, inside the field of HIV and beyond. If nothing else, research into a block-and lock-strategy will provide valuable knowledge on HIV transcription for the future of all HIV cure research.

## References

[1] Finzi D., Hermankova M., Pierson T., Carruth L.M., Buck C., Chaisson R.E., Quinn T.C., Chadwick K., Margolick J., Brookmeyer R. (1997). Identification of a Reservoir for HIV-1 in Patients on Highly Active Antiretroviral Therapy. Science.

[2] Barton K., Winckelmann A., Palmer S. (2016). HIV-1 Reservoirs During Suppressive Therapy. Trends Microbiol..

[3] Finzi D., Blankson J., Siliciano J.D., Margolick J.B., Chadwick K., Pierson T., Smith K., Lisziewica J., Lori F., Flexner C. (1999). Latent infection of CD4+ T cells provides a mechanism for lifelong persistence of HIV-1, even in patients on effective combination therapy. Nat. Med..

[4] Hütter G., Nowak D., Mossner M., Ganepola S., Müßig A., Allers K., Schneider T., Hofmann J., Kücherer C., Blau O. (2009). Long-Term Control of HIV by CCR5 Delta32/Delta32 Stem-Cell Transplantation. N. Engl. J. Med..

[5] Gupta R.K., Abdul-Jawad S., McCoy L.E., Mok H.P., Peppa D., Salgado M., Martinez Piicado J., Nijhuis M., Wensing A.M.J., Lee H. (2019). HIV-1 remission following CCR5Δ32/Δ32 haematopoietic stem-cell transplantation. Nature.

[6] Kordelas L., Verheyen J., Esser S. (2014). Shift of HIV Tropism in Stem-Cell Transplantation with *CCR5* Delta32 Mutation. N. Engl. J. Med..

[7] Verheyen J., Thielen A., Lübke N., Dirks M., Widera M., Dittmer U., Kordelas L., Daumer M., De Jong D.C.M., Wensing A.M.J. (2019). Rapid Rebound of a Preexisting CXCR4-tropic Human Immunodeficiency Virus Variant After Allogeneic Transplantation With CCR5 Δ32 Homozygous Stem Cells. Clin. Infect. Dis..

[8] Henrich T.J., Hanhauser E., Marty F.M., Sirignano M.N., Keating S., Lee T.H., Robles Y.P., Davis B.T., Li J.Z., Heisey A. (2014). Antiretroviral-Free HIV-1 Remission and Viral Rebound After Allogeneic Stem Cell Transplantation. Ann. Intern. Med..

[9] Liao H.K., Gu Y., Diaz A., Marlett J., Takahashi Y., Li M., Suzuki K., Hishida T., Chang C.J., Esteban C.R. (2015). Use of the CRISPR/Cas9 system as an intracellular defense against HIV-1 infection in human cells. Nat. Commun..

[10] Wang G., Zhao N., Berkhout B., Das A.T. (2016). CRISPR-Cas9 Can Inhibit HIV-1 Replication but NHEJ Repair Facilitates Virus Escape. Mol. Ther..

[11] Peterson C.W., Kiem H.-P., Silvestri G., Lichterfeld M. (2018). Cell and Gene Therapy for HIV Cure BT—HIV-1 Latency.

[12] Herrera-Carrillo E., Gao Z., Berkhout B. (2019). CRISPR therapy towards an HIV cure. Brief. Funct. Genomics.

[13] Darcis G., Van Driessche B., Van Lint C. (2016). Preclinical shock strategies to reactivate latent HIV-1: An update. Curr. Opin. HIV AIDS.

[14] Archin N.M., Liberty A.L., Kashuba A.D., Choudhary S.K., Kuruc J.D., Crooks A.M., Parker D.C., Anderson E.M., Kearney M.F., Strain M.C. (2012). Administration of vorinostat disrupts HIV-1 latency in patients on antiretroviral therapy. Nature.

[15] Søgaard O.S., Graversen M.E., Leth S., Olesen R., Brinkmann C.R., Nissen S.K., Kjaer A.S., Schmeimann M.H., Denton P.W., Hey-Cunningham W.J. (2015). The Depsipeptide Romidepsin Reverses HIV-1 Latency In Vivo. PLoS Pathog..

[16] Matalon S., Rasmussen T.A., Dinarello C.A. (2011). Histone deacetylase inhibitors for purging HIV-1 from the latent reservoir. Mol. Med..

[17] Battivelli E., Dahabieh M.S., Abdel-Mohsen M., Svensson J.P., Da Silva I.T., Cohn L.B., Gramatica A., Deeks S., Greene W., Pillai S.K. Chromatin Functional States Correlate with HIV Latency Reversal in Infected Primary CD4+ T Cells. Elife.

[18] Chen H.C., Martinez J.P., Zorita E., Meyerhans A., Filion G.J. (2017). Position effects influence HIV latency reversal. Nat. Struct. Mol. Biol..

[19] Spivak A.M., Planelles V. (2018). Novel Latency Reversal Agents for HIV-1 Cure. Annu. Rev. Med..

[20] Zaikos T.D., Painter M.M., Kettinger N.T.S., Terry V.H., Collins K.L. (2018). Class 1-Selective Histone Deacetylase (HDAC) Inhibitors Enhance HIV Latency Reversal while Preserving the Activity of HDAC Isoforms Necessary for Maximal HIV Gene Expression. J. Virol..

[21] Bouchat S., Delacourt N., Kula A., Darcis G., Van Driessche B., Corazza F., Catot J.S., Melard A., Vanhulle C., Kabeya K. (2016). Sequential treatment with 5-aza-2’-deoxycytidine and deacetylase inhibitors reactivates HIV-1. EMBO Mol. Med..

[22] Shan L., Deng K., Shroff N.S., Durand C.M., Rabi S.A., Yang H.C., Zhang H., Margolick J.B., Blankson J.N., Siliciano R.F. (2012). Stimulation of HIV-1-specific cytolytic T-lymphocytes facilitates elimination of latent viral reservoir after virus reactivation. Immunity.

[23] Kim Y., Anderson J.L., Lewin S.R. (2018). Getting the ‘kill’ into ‘shock and kill’: Strategies to eliminate latent HIV. Cell Host Microbe.

[24] Barouch D.H., Deeks S.G. (2014). Immunologic Strategies for HIV-1 Remission and Eradication. Science.

[25] Macedo A.B., Novis C.L., Bosque A. (2019). Targeting Cellular and Tissue HIV Reservoirs With Toll-Like Receptor Agonists. Front. Immunol..

[26] Wykes M.N., Lewin S.R. (2018). Immune checkpoint blockade in infectious diseases. Nat. Rev. Immunol..

[27] Stephenson K.E. (2018). Therapeutic vaccination for HIV: Hopes and challenges. Curr. Opin. HIV AIDS.

[28] Kumar R., Qureshi H., Deshpande S., Bhattacharya J. (2018). Broadly neutralizing antibodies in HIV-1 treatment and prevention. Ther. Adv. Vaccines Immunother..

[29] Stephenson K.E., Barouch D.H. (2016). Broadly Neutralizing Antibodies for HIV Eradication. Curr. HIV AIDS Rep..

[30] Hattori S.I., Matsuda K., Tsuchiya K., Gatanaga H., Oka S., Yoshimura K., Mitsuya H., Maeda K. (2018). Combination of a Latency-Reversing Agent With a Smac Mimetic Minimizes Secondary HIV-1 Infection in vitro. Front. Microbiol..

[31] Campbell G.R., Bruckman R.S., Chu Y.L., Trout R.N., Spector S.A. (2018). SMAC Mimetics Induce Autophagy-Dependent Apoptosis of HIV-1-Infected Resting Memory CD4+ T Cells. Cell Host Microbe.

[32] Campbell G.R., Spector S.A. (2019). DIABLO/SMAC mimetics selectively kill HIV-1-infected resting memory CD4+ T cells: A potential role in a cure strategy for HIV-1 infection. Autophagy.

[33] Cummins N.W., Sainski-Nguyen A.M., Natesampillai S., Aboulnasr F., Kaufmann S., Badley A.D. (2017). Maintenance of the HIV Reservoir Is Antagonized by Selective BCL2 Inhibition. J. Virol..

[34] Cummins N.W., Sainski A.M., Dai H., Natesampillai S., Pang Y.P., Bren G.D., de Araujo Correia M.C.M., Sampath R., Rizza S.A., O’Brien D. (2016). Prime, Shock, and Kill: Priming CD4 T Cells from HIV Patients with a BCL-2 Antagonist before HIV Reactivation Reduces HIV Reservoir Size. J. Virol..

[35] Lucas A., Kim Y., Rivera-Pabon O., Chae S., Kim D.H., Kim B. (2010). Targeting the PI3K/Akt Cell Survival Pathway to Induce Cell Death of HIV-1 Infected Macrophages with Alkylphospholipid Compounds. PLoS ONE.

[36] Darcis G., Van Driessche B., Van Lint C. (2017). HIV Latency: Should We Shock or Lock?. Trends Immunol..

[37] Pierson T.C., Zhou Y., Kieffer T.L., Ruff C.T., Buck C., Siliciano R.F. (2002). Molecular Characterization of Preintegration Latency in Human Immunodeficiency Virus Type 1 Infection. J. Virol..

[38] Pierson T.C., Kieffer T.L., Ruff C.T., Buck C., Gange S.J., Siliciano R.F. (2002). Intrinsic stability of episomal circles formed during human immunodeficiency virus type 1 replication. J. Virol..

[39] Kelly J., Beddall M.H., Yu D., Iyer S.R., Marsh J.W., Wu Y. (2008). Human macrophages support persistent transcription from unintegrated HIV-1 DNA. Virology.

[40] Pang S., Koyanagi Y., Miles S., Wiley C., Vinters H.V., Chen I.S. (1990). High levels of unintegrated HIV-1 DNA in brain tissue of AIDS dementia patients. Nature.

[41] Roy S., Delling U., Chen C.H., Rosen C.A., Sonenberg N. (1990). A bulge structure in HIV-1 TAR RNA is required for Tat binding and Tat-mediated trans-activation. Genes Dev..

[42] Zhu Y., Pe’ery T., Peng J., Ramanathan Y., Marshall N., Marshall T., Amendit B., Mathews M.B., Price D.H. (1997). Transcription elongation factor P-TEFb is required for HIV-1 Tat transactivation in vitro. Genes Dev..

[43] Ping Y.H., Rana T.M. (2001). DSIF and NELF Interact with RNA Polymerase II Elongation Complex and HIV-1 Tat Stimulates P-TEFb-mediated Phosphorylation of RNA Polymerase II and DSIF during Transcription Elongation. J. Biol. Chem..

[44] Easley R., Carpio L., Dannenberg L., Choi S., Alani D., Van Duyne R., Guendel I., Klase Z., Agbottah E., Kehn-Hall K. (2010). Transcription through the HIV-1 nucleosomes: Effects of the PBAF complex in Tat activated transcription. Virology.

[45] Ammosova T., Berro R., Jerebtsova M., Jackson A., Charles S., Klase Z., Southerland W., Gordeuk V.R., Kashanchi F., Nekhai S. (2006). Phosphorylation of HIV-1 Tat by CDK2 in HIV-1 transcription. Retrovirology.

[46] Yang Z., Zhu Q., Luo K., Zhou Q. (2001). The 7SK small nuclear RNA inhibits the CDK9/cyclin T1 kinase to control transcription. Nature.

[47] Yik J.H., Chen R., Nishimura R., Jennings J.L., Link A.J., Zhou Q. (2003). Inhibition of P-TEFb (CDK9/Cyclin T) Kinase and RNA Polymerase II Transcription by the Coordinated Actions of HEXIM1 and 7SK snRNA. Mol. Cell.

[48] Jang M.K., Mochizuki K., Zhou M., Jeong H.S., Brady J.N., Ozato K. (2005). The Bromodomain Protein Brd4 Is a Positive Regulatory Component of P-TEFb and Stimulates RNA Polymerase II-Dependent Transcription. Mol. Cell.

[49] Bisgrove D.A., Mahmoudi T., Henklein P., Verdin E. (2007). Conserved P-TEFb-interacting domain of BRD4 inhibits HIV transcription. Proc. Natl. Acad. Sci. USA.

[50] West M.J., Lowe A.D., Karn J. (2001). Activation of human immunodeficiency virus transcription in T cells revisited: NF-kappaB p65 stimulates transcriptional elongation. J. Virol..

[51] Hogan P.G., Chen L., Nardone J., Rao A. (2003). Transcriptional regulation by calcium, calcineurin, and NFAT. Genes Dev..

[52] Kinoshita S., Su L., Amano M., Timmerman L.A., Kaneshima H., Nolan G.P. (1997). The T Cell Activation Factor NF-ATc Positively Regulates HIV-1 Replication and Gene Expression in T Cells. Immunity.

[53] Taube R., Peterlin M. (2013). Lost in transcription: Molecular mechanisms that control HIV latency. Viruses.

[54] Delannoy A., Poirier M., Bell B. (2019). Cat and Mouse: HIV Transcription in Latency, Immune Evasion and Cure/Remission Strategies. Viruses.

[55] Mbonye U., Karn J. (2017). The Molecular Basis for Human Immunodeficiency Virus Latency. Annu. Rev. Virol..

[56] Khoury G., Darcis G., Lee M.Y., Bouchat S., Van Driessche B., Purcell D.F.J., Van Lint C., Zhang L., Lewin S.R. (2018). The Molecular Biology of HIV Latency BT—HIV Vaccines and Cure: The Path Towards Finding an Effective Cure and Vaccine.

[57] Coull J.J., Romerio F., Sun J.M., Volker J.L., Galvin K.M., Davie J.R., Shi Y., Hansen U., Margolis D.M. (2000). The Human Factors YY1 and LSF Repress the Human Immunodeficiency Virus Type 1 Long Terminal Repeat via Recruitment of Histone Deacetylase 1. J. Virol..

[58] Tyagi M., Karn J. (2007). CBF-1 promotes transcriptional silencing during the establishment of HIV-1 latency. EMBO J..

[59] He G., Margolis D.M. (2002). Counterregulation of chromatin deacetylation and histone deacetylase occupancy at the integrated promoter of human immunodeficiency virus type 1 (HIV-1) by the HIV-1 repressor YY1 and HIV-1 activator Tat. Mol. Cell. Biol..

[60] Pearson R., Kim Y.K., Hokello J., Lassen K., Friedman J., Tyagi M., Karn J. (2008). Epigenetic Silencing of Human Immunodeficiency Virus (HIV) Transcription by Formation of Restrictive Chromatin Structures at the Viral Long Terminal Repeat Drives the Progressive Entry of HIV into Latency. J. Virol..

[61] Tyagi M., Pearson R.J., Karn J. (2010). Establishment of HIV Latency in Primary CD4+ Cells Is due to Epigenetic Transcriptional Silencing and P-TEFb Restriction. J. Virol..

[62] Krajewski W.A., Becker P.B. (1998). Reconstitution of hyperacetylated, DNase I-sensitive chromatin characterized by high conformational flexibility of nucleosomal DNA. Proc. Natl. Acad. Sci. USA.

[63] Bannister A.J., Schneider R., Myers F.A., Thorne A.W., Crane-Robinson C., Kouzarides T. (2005). Spatial Distribution of Di- and Tri-methyl Lysine 36 of Histone H3 at Active Genes. J. Biol. Chem..

[64] Du Chéné I., Basyuk E., Lin Y.L., Triboulet R., Knezevich A., Chable-Bessia C., Mettling C., Baillat V., Reynes J., Corbeau P. (2007). Suv39H1 and HP1gamma are responsible for chromatin-mediated HIV-1 transcriptional silencing and post-integration latency. EMBO J..

[65] Trejbalová K., Kovářová D., Blažková J., Machala L., Jilich D., Weber J., Kučerová D., Vencalek O., Hirsch I., Hejnar J. (2016). Development of 5’ LTR DNA methylation of latent HIV-1 provirus in cell line models and in long-term-infected individuals. Clin. Epigenetics.

[66] Blazkova J., Trejbalova K., Gondois-Rey F., Halfon P., Philibert P., Guiguen A., Verdin E., Olive D., Van Lint C., Hejnar J. (2009). CpG methylation controls reactivation of HIV from latency. PLoS Pathog..

[67] Verdin E.R.I.C. (1991). DNase I-hypersensitive sites are associated with both long terminal repeats and with the intragenic enhancer of integrated human immunodeficiency virus type 1. J. Virol..

[68] Yukl S.A., Kaiser P., Kim P., Telwatte S., Joshi S.K., Vu M., Lampiris H., Wong J.K. (2018). HIV latency in isolated patient CD4+ T cells may be due to blocks in HIV transcriptional elongation, completion, and splicing. Sci. Transl. Med..

[69] Telwatte S., Lee S., Somsouk M., Hatano H., Baker C., Kaiser P., Kim P., Chen T.H., Milush J., Hunt P.W. (2018). Gut and blood differ in constitutive blocks to HIV transcription, suggesting tissue-specific differences in the mechanisms that govern HIV latency. PLoS Pathog..

[70] Misteli T. (2007). Beyond the Sequence: Cellular Organization of Genome Function. Cell.

[71] Hübner M.R., Spector D.L. (2010). Chromatin dynamics. Annu. Rev. Biophys..

[72] Lusic M., Siliciano R.F. (2016). Nuclear landscape of HIV-1 infection and integration. Nat. Rev. Microbiol..

[73] Debyser Z., Christ F., De Rijck J., Gijsbers R. (2015). Host factors for retroviral integration site selection. Trends Biochem. Sci..

[74] Marini B., Kertesz-Farkas A., Ali H., Lucic B., Lisek K., Manganaro L., Pongor S., Luzzati R., Recchia A., Mavillo F. (2015). Nuclear architecture dictates HIV-1 integration site selection. Nature.

[75] Ciuffi A., Llano M., Poeschla E., Hoffmann C., Leipzig J., Shinn P., Echer J.R., Bushman F. (2005). A role for LEDGF/p75 in targeting HIV DNA integration. Nat. Med..

[76] Vranckx L.S., Demeulemeester J., Saleh S., Boll A., Vansant G., Schrijvers R., Weydert C., Batticelli E., Verdin E., Ceresedo A. (2016). LEDGIN-mediated Inhibition of Integrase–LEDGF/p75 Interaction Reduces Reactivation of Residual Latent HIV. EBioMedicine.

[77] Schrijvers R., Vets S., De Rijck J., Malani N., Bushman F.D., Debyser Z., Gijsbers R. (2012). HRP-2 determines HIV-1 integration site selection in LEDGF/p75 depleted cells. Retrovirology.

[78] Chin C.R., Perreira J.M., Savidis G., Portmann J.M., Aker A.M., Feeley E.M., Smith M.C., Brass A.L. (2015). Direct Visualization of HIV-1 Replication Intermediates Shows that Capsid and CPSF6 Modulate HIV-1 Intra-nuclear Invasion and Integration. Cell Rep..

[79] Rasheedi S., Shun M.C., Serrao E., Sowd G.A., Qian J., Hao C., Dasgupta T., Engelman A.N., Skoronski J. (2016). The Cleavage and Polyadenylation Specificity Factor 6 (CPSF6) Subunit of the Capsid-recruited Pre-messenger RNA Cleavage Factor I (CFIm) Complex Mediates HIV-1 Integration into Genes. J. Biol. Chem..

[80] Achuthan V., Perreira J.M., Sowd G.A., Puray-Chavez M., McDougall W.M., Paulucci-Holthauzen A., Wu X., Fadel H.J., Poeschla E.M., Multani A.S. (2018). Capsid-CPSF6 Interaction Licenses Nuclear HIV-1 Trafficking to Sites of Viral DNA Integration. Cell Host Microbe.

[81] Wagner T.A., McLaughlin S., Garg K., Cheung C.Y., Larsen B.B., Styrchak S., Huang H.C., Edlefsen P.T., Mullins J.I., Frenkel L.M. (2014). Proliferation of cells with HIV integrated into cancer genes contributes to persistent infection. Science.

[82] Maldarelli F., Wu X., Su L., Simonetti F.R., Shao W., Hill S., Spindler J., Ferris A.L., Mellors J.W., Kearney M.F. (2014). Specific HIV integration sites are linked to clonal expansion and persistence of infected cells. Science.

[83] Cohn L.B., Silva I.T., Oliveira T.Y., Rosales R.A., Parrish E.H., Learn G.H., Hahn B.H., Czartoski J.L., McElarch M.J., Lehmann C. (2015). HIV-1 Integration Landscape during Latent and Active Infection. Cell.

[84] Cesana D., de Sio F.R.S., Rudilosso L., Gallina P., Calabria A., Beretta S., Merelli I., Bruzzesi E., Passerini L., Nozza S. (2017). HIV-1-mediated insertional activation of STAT5B and BACH2 trigger viral reservoir in T regulatory cells. Nat. Commun..

[85] Kobayashi S., Taki T., Chinen Y., Tsutsumi Y., Ohshiro M., Kobayashi T., Matsumoto Y., Kuroda J., Horiike S., Nishida K. (2011). Identification of IGHCδ–BACH2 fusion transcripts resulting from cryptic chromosomal rearrangements of 14q32 with 6q15 in aggressive B-cell lymphoma/leukemia. Genes Chromosom. Cancer.

[86] Flucke U., Tops B.B., de Saint Aubain Somerhausen N., Bras J., Creytens D.H., Küsters B., Groenen P.J.T.A., Verdijik M.A.J., Suurmeijier A.J.H., Mentzel T. (2013). Presence of C11orf95–MKL2 fusion is a consistent finding in chondroid lipomas: A study of eight cases. Histopathology.

[87] Rice A.P. (2017). The HIV-1 Tat Protein: Mechanism of Action and Target for HIV-1 Cure Strategies. Curr. Pharm. Des..

[88] Mousseau G., Clementz M.A., Bakeman W.N., Nagarsheth N., Cameron M., Shi J., Baran P., Fromentin R., Chomont N., Valente S.T. (2012). An analog of the natural steroidal alkaloid cortistatin A potently suppresses Tat-dependent HIV transcription. Cell Host Microbe.

[89] Mousseau G., Kessing C.F., Fromentin R., Trautmann L., Chomont N., Valente S.T. (2015). The Tat Inhibitor Didehydro-Cortistatin A Prevents HIV-1 Reactivation from Latency. MBio.

[90] Kessing C.F., Nixon C.C., Li C., Tsai P., Takata H., Mousseau G., Ho P.T., Honeycutt J.B., Fallahi M., Trautmann L. (2017). In vivo suppression of HIV rebound by didehydro-Cortistatin A, a ‘block-and-lock’ strategy for HIV-1 cure. Cell Rep..

[91] Li C., Mousseau G., Valente S.T. (2019). Tat inhibition by didehydro-Cortistatin A promotes heterochromatin formation at the HIV-1 long terminal repeat. Epigenetics Chromatin.

[92] Christ F., Voet A., Marchand A., Nicolet S., Desimmie B.A., Marchand D., Bardiot D., Van der Veken N.J., Van Remoortel B., De Maeyer M. (2010). Rational design of small-molecule inhibitors of the LEDGF/p75-integrase interaction and HIV replication. Nat. Chem. Biol..

[93] Al-Mawsawi L.Q., Neamati N. (2011). Allosteric Inhibitor Development Targeting HIV-1 Integrase. ChemMedChem.

[94] Kessl J.J., Jena N., Koh Y., Taskent-Sezgin H., Slaughter A., Feng L., de Silva S., Wu L., Le Grice S.F.J., Engelman A. (2012). Multimode, Cooperative Mechanism of Action of Allosteric HIV-1 Integrase Inhibitors. J. Biol. Chem..

[95] Tsiang M., Jones G.S., Niedziela-Majka A., Kan E., Lansdon E.B., Huang W., Hung M., Samuel D., Novikov N., Xu Y. (2012). New Class of HIV-1 Integrase (IN) Inhibitors with a Dual Mode of Action. J. Biol. Chem..

[96] Fenwick C., Bailey M.D., Bethell R., Bös M., Bonneau P., Cordingley M., Coulombe R., Duan J., Edwards P., Fader L.D. (2014). Preclinical profile of BI 224436, a novel HIV-1 non-catalytic-site integrase inhibitor. Antimicrob. Agents Chemother..

[97] Christ F., Shaw S., Demeulemeester J., Desimmie B.A., Marchand A., Butler S., Smets W., Chaltin P., Westby M., Debyser Z. (2012). Small-Molecule Inhibitors of the LEDGF/p75 Binding Site of Integrase Block HIV Replication and Modulate Integrase Multimerization. Antimicrob. Agents Chemother..

[98] Desimmie B.A., Schrijvers R., Demeulemeester J., Borrenberghs D., Weydert C., Thys W., Vets S., Van Remoortel B., Hofkens J., Bannert N. (2013). LEDGINs inhibit late stage HIV-1 replication by modulating integrase multimerization in the virions. Retrovirology.

[99] Jurado K.A., Wang H., Slaughter A., Feng L., Kessl J.J., Koh Y., Wang W., Ballandras-Colas A., Patel P.A., Fuchs J.R. (2013). Allosteric integrase inhibitor potency is determined through the inhibition of HIV-1 particle maturation. Proc. Natl. Acad. Sci. USA.

[100] Balakrishnan M., Yant S.R., Tsai L., O’Sullivan C., Bam R.A., Tsai A., Niedziela-Majka A., Stray K.M., Sakowicz R., Cihlar T. (2013). Non-Catalytic Site HIV-1 Integrase Inhibitors Disrupt Core Maturation and Induce a Reverse Transcription Block in Target Cells. PLoS ONE.

[101] Le Rouzic E., Bonnard D., Chasset S., Bruneau J.M., Chevreuil F., Le Strat F., Nguyen J., Beauvoir R., Amadori C., Brias J. (2013). Dual inhibition of HIV-1 replication by integrase-LEDGF allosteric inhibitors is predominant at the post-integration stage. Retrovirology.

[102] Debyser Z., Vansant G., Bruggemans A., Janssens J., Christ F. (2018). Insight in HIV Integration Site Selection Provides a Block-and-Lock Strategy for a Functional Cure of HIV Infection. Viruses.

[103] Vansant G., Vranckx L.S., Zurnic I., Van Looveren D., Van de Velde P., Nobles C., Gijsbers R., Christ F., Debyser Z. (2019). Impact of LEDGIN treatment during virus production on residual HIV-1 transcription. Retrovirology.

[104] UNAIDS (Joint United Nations Programme on HIV/AIDS) (2019). Global HIV & AIDS Statistics: 2019 Fact Sheet.

[105] Orphanides G., Wu W.H., Lane W.S., Hampsey M., Reinberg D. (1999). The chromatin-specific transcription elongation factor FACT comprises human SPT16 and SSRP1 proteins. Nature.

[106] Orphanides G., LeRoy G., Chang C.H., Luse D.S., Reinberg D. (1998). Reinberg, FACT, a Factor that Facilitates Transcript Elongation through Nucleosomes. Cell.

[107] Gasparian A.V., Burkhart C.A., Purmal A.A., Brodsky L., Pal M., Saranadasa M., Bosykh D.A., Commane M., Guryanova O.A., Pal S. (2011). Curaxins: Anticancer compounds that simultaneously suppress NF-κB and activate p53 by targeting FACT. Sci. Transl. Med..

[108] Jean M.J., Hayashi T., Huang H., Brennan J., Simpson S., Purmal A., Gurova K., Keefer M.C., Kobie J.J., Santoso N.G. (2017). Curaxin CBL0100 Blocks HIV-1 Replication and Reactivation through Inhibition of Viral Transcriptional Elongation. Front. Microbiol..

[109] Suzuki K., Juelich T., Lim H., Ishida T., Watanebe T., Cooper D.A., Rao S., Kelleher A.D. (2008). Closed chromatin architecture is induced by an RNA duplex targeting the HIV-1 promoter region. J. Biol. Chem..

[110] Ahlenstiel C., Mendez C., Lim S.T., Marks K., Turville S., Cooper D.A., Kelleher A.D., Suzuki K. (2015). Novel RNA Duplex Locks HIV-1 in a Latent State via Chromatin-mediated Transcriptional Silencing. Mol. Ther. Nucleic Acids.

[111] Méndez C., Ledger S., Petoumenos K., Ahlenstiel C., Kelleher A.D. (2018). RNA-induced epigenetic silencing inhibits HIV-1 reactivation from latency. Retrovirology.

[112] Ringrose J.H., Jeeninga R.E., Berkhout B., Speijer D. (2008). Proteomic studies reveal coordinated changes in T-cell expression patterns upon infection with human immunodeficiency virus type 1. J. Virol..

[113] Boukli N.M., Shetty V., Cubano L., Ricaurte M., Coelho-dos-Reis J., Nickens Z., Shah P., Talal A.H., Philip R., Jain P. (2012). Unique and differential protein signatures within the mononuclear cells of HIV-1 and HCV mono-infected and co-infected patients. Clin. Proteomics.

[114] Vozzolo L., Loh B., Gane P.J., Tribak M., Zhou L., Anderson I., Nyakatura E., Jenner R.G., Selwood D., Fassati A. (2010). Gyrase B inhibitor impairs HIV-1 replication by targeting Hsp90 and the capsid protein. J. Biol. Chem..

[115] Joshi P., Stoddart C.A. (2011). Impaired infectivity of ritonavir-resistant HIV is rescued by heat shock protein 90AB1. J. Biol. Chem..

[116] Anderson I., Low J.S., Weston S., Weinberger M., Zhyvoloup A., Labokha A.A., Corazza G., Kitson R.A., Moody J.C., Marcello A. (2014). Heat shock protein 90 controls HIV-1 reactivation from latency. Proc. Natl. Acad. Sci. USA.

[117] Kim H., Choi M.S., Inn K.S., Kim B.J. (2016). Inhibition of HIV-1 reactivation by a telomerase-derived peptide in a HSP90-dependent manner. Sci. Rep..

[118] Roesch F., Meziane O., Kula A., Nisole S., Porrot F., Anderson I., Mammano F., Fassati A., Marcello A., Benkirane M. (2012). Hyperthermia Stimulates HIV-1 Replication. PLOS Pathog..

[119] Joshi P., Maidji E., Stoddart C.A. (2016). Inhibition of Heat Shock Protein 90 Prevents HIV Rebound. J. Biol. Chem..

[120] Chiang N.J., Yeh K.H., Chiu C.F., Chen J.S., Yen C.C., Lee K.D.L.L., Bai L.Y., Chen M.H., Lin J.S., Yang Y. (2016). Results of Phase II trial of AUY922, a novel heat shock protein inhibitor in patients with metastatic gastrointestinal stromal tumor (GIST) and imatinib and sunitinib therapy. J. Clin. Oncol..

[121] Oki Y., Younes A., Knickerbocker J., Samaniego F., Nastoupil L., Hagemeister F., Romaguera J., Fowler N., Kwak L., Westin J. (2015). Experience with HSP90 inhibitor AUY922 in patients with relapsed or refractory non-Hodgkin lymphoma. Haematologica.

[122] Jhaveri K., Taldone T., Modi S., Chiosis G. (2012). Advances in the clinical development of heat shock protein 90 (Hsp90) inhibitors in cancers. Biochim. Biophys. Acta Mol. Cell Res..

[123] Talaei S., Mellatyar H., Asadi A., Akbarzadeh A., Sheervalilou R., Zarghami N. (2019). Spotlight on 17-AAG as an Hsp90 inhibitor for molecular targeted cancer treatment. Chem. Biol. Drug Des..

[124] Gavegnano C., Detorio M., Montero C., Bosque A., Planelles V., Schinazi R.F. (2014). Ruxolitinib and Tofacitinib Are Potent and Selective Inhibitors of HIV-1 Replication and Virus Reactivation in vitro. Antimicrob. Agents Chemother..

[125] Gavegnano C., Brehm J.H., Dupuy F.P., Talla A., Ribeiro S.P., Kulpa D.A., Cameron C., Santos S., Hurwitz S.J., Marconi V.C. (2017). Novel mechanisms to inhibit HIV reservoir seeding using Jak inhibitors. PLoS Pathog..

[126] Yang Z., Yik J.H., Chen R., He N., Jang M.K., Ozato K., Zhou Q. (2005). Recruitment of P-TEFb for Stimulation of Transcriptional Elongation by the Bromodomain Protein Brd4. Mol. Cell.

[127] Niu Q., Liu Z., Alamer E., Fan X., Chen H., Endsley J., Gelman B.B., Tian B., Kim J.H., Michael N.L. (2019). Structure-guided drug design identifies a BRD4-selective small molecule that suppresses HIV. J. Clin. Investg..

[128] Besnard E., Hakre S., Kampmann M., Lim H.W., Hosmane N.N., Martin A., Bassik M.C., Verschueren E., Battivelli E., Chan J. (2016). The mTOR complex controls HIV Latency. Cell Host Microbe.

[129] Giacca M. (2016). HIV Latency TORn Down. Cell Host Microbe.

[130] Kampmann M., Bassik M.C., Weissman J.S. (2013). Integrated platform for genome-wide screening and construction of high-density genetic interaction maps in mammalian cells. Proc. Natl. Acad. Sci. USA.

[131] Vargas B., Giacobbi N.S., Sanyal A., Venkatachari N.J., Han F., Gupta P., Sluis-Cremer N. (2019). Inhibitors of Signaling Pathways That Block Reversal of HIV-1 Latency. Antimicrob. Agents Chemother..

[132] Liu Q. (2011). Triptolide and its expanding multiple pharmacological functions. Int. Immunopharmacol..

[133] Wan Z., Chen X. (2014). Triptolide inhibits human immunodeficiency virus type 1 replication by promoting proteasomal degradation of Tat protein. Retrovirology.

[134] Baxter A.E., O’Doherty U., Kaufmann D.E. (2018). Beyond the replication-competent HIV reservoir: Transcription and translation-competent reservoirs. Retrovirology.

[135] Paiardini M., Müller-Trutwin M. (2013). HIV-associated chronic immune activation. Immunol. Rev..

[136] Imamichi H., Dewar R.L., Adelsberger J.W., Rehm C.A., O’Doherty U., Paxinos E.E., Fauci A.S., Lane H.C. (2016). Defective HIV-1 proviruses produce novel protein-coding RNA species in HIV-infected patients on combination antiretroviral therapy. Proc. Natl. Acad. Sci. USA.

[137] Garcia-Tellez T., Huot N., Ploquin M.J., Rascle P., Jacquelin B., Müller-Trutwin M. (2016). Non-human primates in HIV research: Achievements, limits and alternatives. Infect. Genet. Evol..

[138] Jern P., Coffin J.M. (2008). Effects of Retroviruses on Host Genome Function. Annu. Rev. Genet..

[139] Mediouni S., Jablonski J., Paris J.J., Clementz M.A., Thenin-Houssier S., McLaughlin J.P., Valente S.T. (2015). Didehydro-Cortistatin A Inhibits HIV-1 Tat Mediated Neuroinflammation and Prevents Potentiation of Cocaine Reward in Tat Transgenic Mice. Curr. HIV Res..

[140] Mousseau G., Aneja R., Clementz M.A., Mediouni S., Lima N.S., Haregot A., Kessing C.F., Jablonski J.A., Thenin-Houssier S., Nagarsheth N. (2019). Resistance to the tat inhibitor didehydro-cortistatin a is mediated by heightened basal HIV-1 transcription. MBio.

[141] Wolfe L. (2016). Ruxolitinib in Myelofibrosis and Polycythemia Vera. J. Adv. Pract. Oncol..

[142] Winthrop K.L. (2017). The emerging safety profile of JAK inhibitors in rheumatic disease. Nat. Rev. Rheumatol..

[143] Hasinoff B.B., Patel D. (2010). The lack of target specificity of small molecule anticancer kinase inhibitors is correlated with their ability to damage myocytes in vitro. Toxicol. Appl. Pharmacol..

[144] Liu J., Jiang Z., Liu L., Zhang Y., Zhang S., Xiao J., Ma M., Zhang L. (2011). Triptolide induces adverse effect on reproductive parameters of female Sprague-Dawley rats. Drug Chem. Toxicol..

[145] Fabian M.A., Biggs III W.H., Treiber D.K., Atteridge C.E., Azimioara M.D., Benedetti M.G., Carter T.A., Ciceri P., Edeen P.T., Floyd M. (2005). A small molecule–kinase interaction map for clinical kinase inhibitors. Nat. Biotechnol..

